# Applying mixed methods to pilot feasibility studies to inform intervention trials

**DOI:** 10.1186/s40814-022-01178-x

**Published:** 2022-09-26

**Authors:** Kelly A. Aschbrenner, Gina Kruse, Joseph J. Gallo, Vicki L. Plano Clark

**Affiliations:** 1 Psychiatry, Geisel School of Medicine at Dartmouth College, Hanover, USA; 2 Division of General Internal Medicine, Massachusetts General Hospital, Boston, USA; 3 Department of Mental Health, Bloomberg School of Public Health, Johns Hopkins University, Baltimore, USA; 4 School of Education Research Methods, University of Cincinnati, Cincinnati, USA

**Keywords:** Mixed methods, Pilot studies, Intervention, Feasibility studies, Integration, Quantitative and qualitative, Methodological guidance

## Abstract

**Background:**

Pilot feasibility studies serve a uniquely important role in preparing for larger scale intervention trials by examining the feasibility and acceptability of interventions and the methods used to test them. Mixed methods (collecting, analyzing, and integrating quantitative and qualitative data and results) can optimize what can be learned from pilot feasibility studies to prepare rigorous intervention trials. Despite increasing use of mixed method designs in intervention trials, there is limited guidance on how to apply these approaches to address pilot feasibility study goals. The purpose of this article is to offer methodological guidance for how investigators can plan to integrate quantitative and qualitative methods within pilot feasibility studies to comprehensively address key research questions.

**Methods:**

We used an informal consensus-based process informed by key methodological resources and our team’s complementary expertise as intervention researchers and mixed methodologists to develop guidance for applying mixed methods to optimize what can be learned from pilot feasibility studies. We developed this methodological guidance as faculty in the Mixed Methods Research Training Program (MMRTP) for the Health Sciences (R25MH104660) funded by the National Institutes of Health through the Office of Behavioral and Social Science Research.

**Results:**

We provide the following guidance for applying mixed methods to optimize pilot feasibility studies: (1) identify feasibility domain(s) that will be examined using mixed methods, (2) align quantitative and qualitative data sources for the domain(s) selected for mixing methods, (3) determine the timing of the quantitative and qualitative data collection within the flow of the pilot study, (4) plan integrative analyses using joint displays to understand feasibility, and (5) prepare to draw meta-inferences about feasibility and implications for the future trial from the integrated data.

**Conclusions:**

By effectively integrating quantitative and qualitative data within pilot feasibility studies, investigators can harness the potential of mixed methods for developing comprehensive and nuanced understandings about feasibility. Our guidance can help researchers to consider the range of key decisions needed during intervention pilot feasibility testing to achieve a rigorous mixed methods approach generating enhanced insights to inform future intervention trials.

**Supplementary Information:**

The online version contains supplementary material available at 10.1186/s40814-022-01178-x.

## Key messages regarding feasibility

- Despite increasing use of mixed method designs in intervention trials, there is limited guidance on how to apply these approaches to address pilot feasibility study goals.
- We provide guidance for applying mixed methods to optimize pilot feasibility studies.
- Our guidance can help researchers to consider the range of key decisions needed during intervention pilot feasibility testing to achieve a rigorous mixed methods approach generating enhanced insights to inform design of intervention trials.

## Background

Intervention research in the health sciences encompasses the procedures used to develop, refine, and test the efficacy and effectiveness of an intervention on a clinical outcome [1, 2]. Intervention development is often an iterative, nonlinear process of piloting a version of an intervention, getting feedback from participants and collaborators to identify problems, implementing solutions to address problems, and repeating this cycle until the intervention and study procedures are determined to be feasible and acceptable [3, 4]. Pilot feasibility studies occur during intervention development or adaptation and are critical for informing decisions about whether and how to design rigorous efficacy and effectiveness trials [5]. The primary aim of pilot feasibility studies is to examine the feasibility of interventions and the methods used to evaluate them to answer the overarching question “Can it work?” prior to examining “Does it work?” [6].

While pilot feasibility studies are critical components of intervention development, adaptation, and testing [7–9], they can be challenging to design, implement, and interpret. Topic areas commonly addressed by pilot feasibility studies include acceptability, usability, appropriateness, practicality, adaptation, and implementation barriers and facilitators [10]. Within these areas, investigators focus on specific domains of feasibility including recruitment capability, randomization acceptability, data collection procedures and outcome measures, intervention delivery and participant acceptability, intervention adherence and safety, barriers and facilitators to implementing the study, and retention of participants in both the treatment and study [4, 11]. Examples of feasibility domains and brief definitions are listed in Table 1. Many factors can affect these feasibility domains, including providers’ and other professionals’ willingness and ability to assist with recruitment, participants’ time, capacity, and interest in completing assessments and participating in the intervention and whether the research team has the expertise, skills, space, and time to conduct the study [4]. Pilot feasibility testing becomes more complex when research is conducted with populations historically underrepresented in clinical trials [12, 13] and in low-resource settings that present significant organizational, cultural, and infrastructure challenges [14].

**Table 1:** Example feasible domains and brief definitions in pilot feasibility studies

Feasibility domains	Definitions
Recruitment capability	The extent to which study recruitment goals are met
Randomization	The acceptability of randomization and related procedures
Retention	The extent to which participants are enrolled in a trial for the duration of the study
Assessment procedures	The extent to which study assessments can be administered as planned; assessment procedures are acceptable to participants; and data collection is complete
Implementation resources	The adequacy of resources, including time and costs, required to deliver an intervention and conduct the overall study
Intervention delivery, adherence, and safety	The extent to which an intervention is delivered as intended (i.e., fidelity); participants’ behavior corresponds with intervention recommendations (i.e., adherence) and can safely be performed (i.e., safety)
Acceptability	The extent to which an intervention; components of an intervention; and/or study conditions, including a waitlist condition, usual care, or inactive control condition, are satisfactory

This table does not provide a comprehensive list of key domains in pilot feasibility studies. Investigators may choose to include fewer feasibility domains or add or modify domains depending on research questions and contexts

Investigators often use pre-specified criteria to evaluate feasibility domains and determine whether or how to proceed with a future trial [15]; however, there has been limited guidance on what should be considered when formulating progression criteria [15]. Investigators commonly use quantitative metrics such as recruitment and retention rates to determine whether or not pre-specified feasibility criteria and milestones were met to signal that the research can advance to the next stage of testing [7, 13, 16]. However, applying binary indicators of feasibility provides limited information about why aspects of intervention or study procedures were or were not feasible and what improvements are needed to enhance feasibility [17].

Qualitative methods have been used within pilot studies to explore aspects of feasibility in more depth and from the perspective of key stakeholders, including individuals and organizations that have an interest in or are affected by the intervention [18]. O’Cathain and colleagues [19] provided methodological guidance for using a variety of qualitative methods alongside quantitative approaches in feasibility studies to explore uncertainties and optimize an intervention or trial procedures before conducting a fully powered trial. Despite the potential value of combining quantitative and qualitative methods to better capture the complexity of interventions and implementation contexts [20, 21], the use of more than one method further adds to the challenge of implementing and interpreting pilot feasibility studies.

Since O’Cathain and colleagues [19] published their guidance, there have been calls for intervention researchers to not only use quantitative and qualitative methods but to meaningfully integrate them within rigorous mixed methods approaches [22, 23].

This article extends previous literature by offering practical guidance for how investigators can plan to integrate quantitative and qualitative data within pilot feasibility studies to address key feasibility questions. The authors developed this guidance as faculty in the Mixed Methods Research Training Program (MMRTP) for the Health Sciences (R25MH104660). Our team was motivated to develop and disseminate guidance for applying mixed methods in pilot feasibility studies by observing that while many MMRTP scholars grasped the application of mixed methods in large-scale randomized controlled trials, they had difficulty effectively applying mixed methods designs to small-scale intervention development studies and expressed uncertainty about how to design for data integration in pilot studies given the challenges of numerous feasibility domains of interest. This article was developed in response to requests from MMRTP scholars and other investigators for practical guidance on this topic.

We used an informal consensus-based approach [24] in which all members of our team had equal input on and approved the methodological guidance we generated for applying mixed methods in pilot feasibility studies. Key methodological resources on pilot feasibility studies and mixed methods study designs combined with our team’s complementary experiences and expertise as intervention researchers and mixed methodologists informed the development of our guidance. Methodological resources were reviewed to identify considerations and advice for how to apply mixed methods within the context of intervention research and to identify the primary strategies recommended for achieving integration across mixed methods approaches.

We begin with a brief overview of mixed methods intervention research and then provide methodological guidance for considerations needed to apply mixed methods research in the context of pilot feasibility studies. We emphasize the importance of selecting, among many possible domains, the key domain(s) of feasibility that would benefit most from a mixed methods investigation as a way to help investigators stay focused and use limited resources efficiently in pilot feasibility studies. Specifically, we offer practical guidance for five planning considerations for using mixed methods in pilot feasibility studies, including recommended steps in the planning process and common pitfalls to avoid.

## Brief overview of mixed methods intervention research

Mixed methods research is research in which the investigator intentionally integrates the questions, data sources, analysis procedures, and interpretations associated with both quantitative and qualitative research within a study or program of research [20, 25, 26]. The basic premise of mixed methods research is that the combination of quantitative results about magnitudes and relationships with qualitative results about experiences and meaning can produce enhanced insights about problems of interest [25]. Therefore, the intent for using a mixed methods approach is to generate insights that are more comprehensive, nuanced, contextually situated, and/or valid than could be achieved with a single approach [27, 28]. Mixed methods are particularly relevant for addressing complex problems in health sciences to capture the perspectives of key stakeholders including patients, providers, and organizations [29, 30].

The relative timing of the quantitative and qualitative methods is an important decision when designing and conducting mixed methods studies [27, 28]. For example, quantitative and qualitative methods can be used with sequential timing, with one approach building on the insights obtained from the other. An *explanatory* sequential approach involves the collection and analysis of quantitative data connected to a subsequent collection and analysis of in-depth qualitative data to explain or expand on the initial quantitative results. An *exploratory* sequential approach involves the collection and analysis of qualitative data that builds to a subsequent collection and analysis of quantitative data to assess or generalize the initial qualitative results. Alternatively, quantitative and qualitative methods can be implemented with concurrent timing. In a *convergent* approach, investigators collect and analyze quantitative and qualitative data during the same phase of the research and then merge the two sets of results to create a comprehensive or corroborated interpretation.

A central methodological consideration for all mixed methods approaches is integration, which occurs when investigators link the quantitative and qualitative aspects of a study to each other in ways that produce enhanced insights [27, 31, 32]. There are three common conceptualizations of mixed methods integration [31]. *Connecting* integration occurs when investigators use the results of one method to inform sampling decisions for another method (e.g., selecting interview participants based on quantitative response patterns). *Building* integration occurs when investigators use the results of one method to inform data collection procedures for another method (e.g., developing survey items based on qualitative themes and participant quotes). *Merging* integration occurs when investigators co-analyze, compare, or relate quantitative and qualitative data and results with each other (e.g., comparing quantitative statistical results with qualitative thematic perspectives). Achieving meaningful integration through connecting, building, and/or merging allows investigators to harness the full potential of a mixed methods approach but also represents the fundamental challenge of applying mixed methods research [33]. Numerous techniques have been developed to support integration in mixed methods studies from the design of research questions to the use of integrative analytic procedures [27, 31, 34, 35], but to date, the application of these techniques has been limited.

Mixed methods research can be applied to design rigorous methodologically sound intervention trials, including efficacy trials, effectiveness trials, and effectiveness-implementation hybrid trials that blend design components of clinical effectiveness and implementation research [21, 36, 37]. Fetters and Molina-Azorin [22] advocated that “the modus operandi for conducting interventional studies should be using a mixed methods approach.” By adding qualitative data collection to quantitative assessments of the intervention process and outcomes and intentionally integrating the two approaches, intervention studies become mixed methods research designs [27]. Qualitative data can be collected before, during, and after implementation of the intervention, and integrating the quantitative and qualitative data can help investigators understand not only whether an intervention works but also how and why or why not [22, 36]. Common applications of mixed methods in randomized controlled trials (RCTs) involve embedding qualitative research to explore barriers and facilitators to study recruitment [38, 39], intervention adherence [40, 41], and study retention [42, 43]. Qualitative research has also been used in RCTs to examine mechanisms [44] and contextual influences on interventions and outcomes [45].

We define *mixed methods pilot feasibility studies* as studies in which the investigators intentionally integrate quantitative and qualitative approaches to examine questions about the feasibility and acceptability of the intervention and study procedures from the perspectives of one or more key stakeholders. Similar to mixed methods intervention trials, the fundamental assumption of this approach is that mixing methods can help investigators better understand key questions central to the goals of pilot feasibility research.

## Five planning considerations to optimize mixed methods pilot feasibility studies

In the sections that follow and summarized in Table 2, we provide guidance for five planning considerations that address fundamental questions for designing mixed methods pilot feasibility studies. These planning considerations draw from key methodological literature on (a) pilot study designs [4, 6, 19, 46], (b) the use of mixed methods in intervention research [22, 36, 47], and (c) mixed methods integration strategies [27, 31, 48]. We provide information about each consideration including an overview and recommended steps for applying them to a mixed methods pilot feasibility study. Although we present the considerations in discrete and linear steps, investigators should keep in mind that study planning is a complex and iterative process, and planning considerations should be selected and applied in an order that is most appropriate for a particular pilot feasibility study.

**Table 2:** Mixed methods planning considerations for pilot feasibility studies

Mixed methods planning consideration for pilot feasibility studies	Recommended steps
I. Identify the feasibility domain(s) to examine with mixed methods	1. Identify the feasibility domains of primary concern 2. Focus the plan to mix methods on the feasibility domain(s) of most uncertainty or complexity and/or potential to generate new knowledge that will inform the future trial 3. For the domain(s) needing mixed methods, formulate mixed methods integration questions consistent with the reason for wanting to combine quantitative and qualitative information
II. Align quantitative and qualitative data sources for the feasibility domain(s) selected for mixing methods	1. Specify benchmarks and set progression criteria for determining feasibility for the selected domain(s) 2. Identify the most relevant participants for the selected feasibility domain(s) 3. Identify the quantitative and qualitative data sources most appropriate for addressing the study’s questions about feasibility and determining whether benchmarks are met 4. Develop a data sources table that indicates the planned participants, data sources, and benchmarks for the examined feasibility domain(s)
III. Determine the timing of the quantitative and qualitative data collection within the flow of the pilot feasibility study activities	1. Map the flow of the major activities in the pilot study 2. Identify the specific points in the diagram when the different quantitative and qualitative data sources will be gathered 3. Apply mixed methods thinking while planning the timing of data collection by considering how the different sources of data can relate to each other 4. Consider how the data collection activities for the data sources relate to the intervention development and pilot study process such as using an iterative approach to piloting and being mindful not to introduce confounding influences that might interfere with the intervention implementation
IV. Plan integrative analyses using joint displays to understand feasibility	1. Plan to develop joint displays about the feasibility domain(s) selected for mixing and the reasons/questions that called for mixing methods 2. Develop a comparison joint display for the reason of triangulation and to corroborate whether a study is feasible for the selected domains 3. Develop a synthesis joint display for the reason of completeness and to form a comprehensive understanding of the complexity of selected feasibility domain(s) 4. Develop an interconnection joint display for the reason of explanation to uncover and explain differential patterns within selected feasibility domain(s) and within different contexts
V. Prepare to draw meta-inferences and implications about feasibility from the integrated data	1. Interpret the quantitative, qualitative, and mixed methods results to draw conclusions and meta-inferences about feasibility 2. Consider the implications for improving the intervention parameters 3. Consider the implications for optimizing the outcome measures 4. Consider the implications for modifying the trial methodology 5. Maintain an audit trail of insights, evidence, and specific implications

### Planning consideration 1: identify the feasibility domain(s) to examine with mixed methods

In the context of intervention development research, several domains of feasibility are often of interest [46, 49]. To maximize the use of mixed methods, *we recommend that investigators identify the domain(s) of feasibility where there is the most uncertainty and/or potential to generate new knowledge that will inform the design of a future intervention trial*. Table 1 provides examples of key feasibility domains of interest. Examples of uncertainty related to these domains include feasibility of online recruitment procedures for populations with limited access to and/or experience using technology, comprehension of randomization among people of lower socioeconomic backgrounds, data collection procedures that have not been previously tested with the target population, and adherence to in-person treatment to a population or in a setting with limited transportation. Not every feasibility domain may require mixing quantitative and qualitative methods and researchers risk unnecessarily complicating pilot study designs with a broad and unfocused application of mixed methods.

Once the feasibility domain(s) of interest are identified, investigators should state their reasons for planning to integrate mixed methods within their pilot feasibility studies. Three common reasons for mixing methods are presented on Table 3 and include the following: *triangulation* (to identify areas of corroboration and dissonance in the data by comparing quantitative and qualitative data), *completeness* (to gain a comprehensive understanding by synthesizing quantitative and qualitative information), and *explanation* (to explain results by connecting quantitative and qualitative information) [50, 51]. A useful strategy for conceptualizing a mixing reason is for investigators to specify a question that will require both quantitative and qualitative data and aligns with the particular reason [27, 52]. For example, “What is the recruitment rate (quantitative question) and what are the barriers and facilitators to recruitment (qualitative question)?” and “How do opinions about the intervention format (qualitative question) differ among participants with high vs. low levels of satisfaction with the intervention (quantitative question)?” By identifying specific questions that call for integrating the data in pilot feasibility studies, investigators can be intentional about their reasons for planning to mix methods within their study. See Table 3 for examples of different mixed methods integration questions about feasibility. A potential pitfall is for investigators to feel compelled to ask mixed methods questions about all feasibility domains, thereby increasing the scope of the pilot study beyond available resources. Instead, investigators should note that some domains may be best addressed with questions that call for quantitative methods or qualitative methods.

**Table 3:** Reasons and questions that call for mixing methods to examine feasibility domains

Common reasons for mixing methods	Example mixed methods data integration questions	
	General examples	Specific examples
***Triangulation***: need to compare quantitative and qualitative evidence to identify areas of corroboration and dissonance to inform a robust decision about feasibility	To what extent and in what ways is (domain of concern) feasible?	How do participant ratings of intervention acceptability compare to what they say about their satisfaction with the intervention?
***Completeness***: need to synthesize quantitative and qualitative information about different facets of feasibility to develop a comprehensive understanding of feasibility	What is the feasibility in terms of (domain of concern) and what barriers need to be addressed?	What recruitment barriers are identified when recruitment rates are combined with participants’ experience of the recruitment process?
***Explanation***: need to interconnect quantitative and qualitative information to uncover and explain differences in feasibility for subgroups or contexts	Does (domain of concern) differ for (subgroups or contexts of interest) and, if so, why?	What are intervention fidelity ratings by study site and why are ratings high, average, and/or low?

Reasons for mixing methods based on “Bryman A. Integrating quantitative and qualitative research: how is it done? Qualitative Research. 2006; 6(1):97-113” and “Greene JC, Caracelli VJ, Graham WF. Toward a Conceptual Framework for Mixed-Method Evaluation Designs. Educational Evaluation and Policy Analysis. 1989; 11(3):255-74”

The following steps are recommended for identifying the feasibility domains that will be examined with and reasons for mixed methods:

1. Identify the feasibility domains of primary concern in the pilot study considering the current stage of development and existing knowledge about the intervention and trial methods.
2. Focus the plan to mix methods on the feasibility domain(s) where there is the most uncertainty regarding feasibility or complexity of the intervention or study procedures and/or potential to generate new knowledge that will inform future intervention trial design. Consider where a combination of methods could potentially provide additional insight and information needed to fully understand feasibility for reasons such as triangulation, completeness, or explanation. One or more domains can be selected for mixing depending on factors like pilot feasibility study timeline and resources.
3. For the domain(s) needing mixed methods, formulate mixed methods integration question(s) consistent with the reason for wanting to combine quantitative and qualitative information to understand and optimize feasibility. For example, if a comprehensive understanding of the recruitment domain is needed, one could ask whether recruitment procedures are feasible as planned and how they can be optimized for study contexts prior to a larger trial.

### Planning consideration 2: align quantitative and qualitative data sources for the feasibility domain(s) selected for mixing methods

The second mixed methods planning consideration involves identifying and aligning the quantitative and qualitative data sources that will be used to address the mixed methods study questions [32, 53]. Investigators using mixed methods need to plan what data will be collected and from whom to support integration of quantitative and qualitative data. To facilitate this process within mixed method approaches, investigators are encouraged to develop data source tables that specify the different data sources (quantitative and/or qualitative) that will provide information about the feasibility domain(s) selected for mixing in the study [20, 54]. See Table 4 for an example of a data source table for mixed methods pilot feasibility studies. Such a table usually includes feasibility domain(s) in the rows and columns for the planned quantitative methods and qualitative methods. This organization assists the investigator with ensuring that both quantitative and qualitative information will be gathered to address the study’s mixed methods questions.

**Table 4:** Example data source table for selected domains in a mixed methods pilot feasibility study

	**Quantitative data**	**Qualitative data**
Recruitment	• Number of participants enrolled per month • Target: 5 enrolled per month • Source: web-based project management software	• Barriers and facilitators to recruitment collected from (a) field notes kept by the study coordinator and (b) interviews with eligible individuals who decided not to participate
Retention	• Number of participants who remained in the study throughout the project period as a proportion of the total number of participants recruited at baseline • Target: 80% retention rate Source: web-based project management software	• Reasons for dropout collected from (a) field notes kept by the study coordinator and (b) interviews with participants who dropped out of the treatment but remain in the study prior to the final assessment
Intervention fidelity	• Degree to which the intervention was delivered as intended • Target: 80% or greater fidelity score • Source: 20-item fidelity measure	• Barriers and facilitators to delivering the intervention collected from (a) on-site observation field notes and (b) weekly supervision notes

In a mixed methods pilot feasibility study, the quantitative and qualitative data sources need to be aligned not only to the feasibility domain(s) selected for mixing but also with pre-specified criteria used to evaluate feasibility. The CONSORT extension states that “a decision process about how to proceed needs to be built into the design of the pilot trial” [15]. Some investigators find it helpful to use a traffic light system (stop-amend-go/red-amber-green) for evaluating progression to a main trial determined by a set of a priori criteria [55, 56]. Examples of progression criteria from pilot trial to a fully powered intervention trial include achieving a pre-specified rate of recruitment in a given time frame, retention or data completion, and levels of intervention acceptability from the perspective of participants [5]. Central to this approach is pausing to amend or refine the intervention and/or study procedures to meet progression criteria before advancing to next steps in the research. Published guidance is available to help investigators set progression criteria for a pilot feasibility study [7, 55, 57, 58].

Once feasibility domains selected for mixing methods have been specified and progression criteria set, investigators should decide which participants and other stakeholders should be included to provide the information necessary to examine the feasibility domains. Individuals who might be important to collect data from are enrolled participants, nonresponders, participants who drop out of the treatment and/or study, caregivers, nurses, physicians, clinic staff, and community members. For data sources, investigators should consider the full range of possible quantitative and qualitative methods to address their feasibility questions and progression criteria [19].

A common pitfall in mixed methods pilot studies is for investigators to default to a “usual” qualitative method, such as focus groups and interviews, without full consideration of the available options. Qualitative methods might include open-ended items on questionnaires, one-on-one interviews, focus groups, unstructured observations, field notes, session recordings, and photographs [59]. With all these options, another potential pitfall for mixed methods pilot feasibility studies occurs when investigators try to gather too much data from too many stakeholders, going beyond their resources and ability to manage it all. Thus, investigators should focus on gathering the data needed to answer the stated feasibility questions and evaluate progression criteria, keeping their resources and the associated ethical considerations in mind.

We recommend the following steps for aligning the data sources needed to address the feasibility domains of interest and plan for integration in a mixed methods pilot study:

1. Specify benchmarks and set clear progression criteria for determining feasibility for the domain(s) of interest.
2. Identify the most relevant participants for the selected feasibility domain(s). Consider who can best contribute to understanding the feasibility concerns, such as enrolled participants, nonresponders, participants who drop out of the treatment and/or study, caregivers, recruiters, intervention clinicians, clinic staff, and community members.
3. Identify the quantitative and qualitative data sources most appropriate for addressing the study’s questions about feasibility and determining whether benchmarks are met. Consider the full range of possible quantitative and qualitative methods and make decisions based on what needs to be learned and the study resources. Keep in mind that even a small sample or data in the form of observation field notes can provide useful information about feasibility.
4. Develop a data sources table that indicates which participants and data sources will provide information for each feasibility domain. Be clear as to which sources are considered quantitative and which are qualitative to see how they align to the study goals and corresponding research questions. Consider adding information about feasibility progression criteria and benchmarks for each domain as well.

### Planning consideration 3: determine the timing of the quantitative and qualitative data collection within the flow of the pilot feasibility study activities

Mixed methods pilot feasibility studies commonly use concurrent mixed methods timing where quantitative and qualitative datasets are collected during the pilot and then analyzed and interpreted together after the intervention is piloted [27, 36]. However, investigators have many options to consider regarding when to collect the quantitative and qualitative data sources in relation to each other and in relation to the flow of activities of the pilot study. To work through decisions about when to collect different data sources, investigators can draw diagrams of their study procedures to plan the flow of the quantitative and qualitative research activities [27, 54]. For a mixed methods pilot study, such a diagram could be organized broadly in terms of activities occurring before, during, and after piloting the intervention [36] or more specifically following the phases of a pilot outlined in the CONSORT extension for pilot and feasibility trials flow diagram (e.g., screening, enrollment, allocation, and assessment) [15]. Investigators should consider the many options for when data could be gathered and determine the most appropriate points within the flow of activities to collect each type of data to optimize learning about the feasibility domain(s) selected for mixing in a pilot feasibility study.

In our experience, it is important to give special attention to the timing of the qualitative data collection. It is common for investigators to wait to gather the qualitative data until the end of a trial. While this approach may have strengths for investigating some feasibility questions, it can have limitations for other feasibility domains such as questions about recruitment, randomization, or retention. For example, to understand why participants withdraw from an intervention after allocation, investigators may want to interview participants soon after the event. Investigators can also plan to implement a flexible approach with regard to timing where qualitative data is collected as problems and implementation barriers arise so they may document information in real time that will help them make necessary refinements to the intervention and study protocol. Investigators might also plan more than one iteration of the pilot study where refinements are made based on initial learnings and then additional data is collected during a subsequent iteration.

The following steps are recommended for planning the timing of the different forms of data collection within the flow of a mixed methods pilot study:

1. Map out the flow of the major activities in the pilot study (e.g., recruitment, intervention, assessment). Some investigators may prefer to use the CONSORT flow diagram (http://www.consort-statement.org/extensions/overview/pilotandfeasibility) for this map, while others prefer to develop their own diagram using shapes and arrows.
2. Within the flow diagram, identify the specific points when the different quantitative and qualitative data sources could be gathered to maximize learning about the feasibility domain(s) selected for mixing.
3. Consider how the different sources of data can relate to each other. Two possibilities include the following:
  a Combine data by gathering both quantitative and qualitative data during the same stages of the pilot. For example, gather recruitment rates alongside field notes about recruiters’ interactions with potential participants and people making the referrals or gather qualitative observations during intervention sessions along with quantitative satisfaction surveys. Using a concurrent approach, the quantitative data on recruitment rates can be merged with qualitative observations to create a comprehensive or corroborated interpretation.
  b Link data by using information from one data source to make decisions about the sample for the other data source. For example, group participants by quantitative adherence scores (high vs. low) and select individuals from each group to interview qualitatively. Using a sequential approach, the quantitative results on adherence can be connected to the qualitative data collection and results to generate explanation and produce enhanced insights
4. Consider how the data collection activities relate to the intervention development and pilot study process. Early in the intervention development, recognize the value of an iterative approach to piloting where initial findings are used to improve the intervention and procedures before piloting again. In the later stages of development, be mindful that the timing of data collection interactions do not introduce bias through confounding influences that might interfere with the implementation of the intervention and raise validity concerns. For example, incorporating qualitative research methods that require additional participation in study activities may have an adverse effect on retention in the trial where additional commitments are required from participants.

### Planning consideration 4: plan integrative analyses using joint displays to understand feasibility

The fourth planning consideration involves envisioning how to bring the quantitative and qualitative data, results, and interpretations together for joint interpretation using integrative analyses. There are a variety of approaches available for conducting integrative analyses [31, 34, 35]. One specific approach that has gained prominence within mixed methods is to develop joint displays [27]. Joint displays are tables (or figures) used to compare, synthesize, or interconnect quantitative and qualitative data, results, and/or interpretations to generate further insights [48]. While these displays can be effective for communicating integrated results within presentations or publications, they are also important analytic tools for investigators to bring the different strands of data together to generate new insights [54]. By developing joint displays, investigators are performing integrative data analyses. See Additional file 1 for template examples of joint displays and citations to published examples for the mixing reasons listed in Table 3.

A potential pitfall is for investigators to focus only on comparing quantitative and qualitative results for agreement without considering if and how to synthesize or interconnect the data. Comparing quantitative and qualitative results in a joint display aligns with triangulation, but as highlighted previously in Table 3, there are multiple reasons why investigators may want to bring together quantitative and qualitative results. For example, an investigator who seeks to explain differences in the feasibility of recruitment across clinics might plan to develop a table where each clinic site is a row and the columns summarize key results for each setting (e.g., quantitative monthly recruitment rates, qualitative barriers and facilitators identified from fieldnotes, and qualitative themes about cultural understandings from clinic partners). By arraying the different results by setting, the investigator might uncover contextual parameters related to recruitment and identify potential modifications to procedures to enhance the feasibility of recruitment.

Investigators wanting to plan integrative analyses using joint displays within their pilot feasibility studies are encouraged to use the following steps:

1. Review the feasibility domain(s) and the mixed methods questions that were asked and the different forms of data and results that are available. Plan to develop joint displays about the feasibility domain(s) selected for mixing and the reasons/questions that called for mixing methods.
2. For a mixing reason of triangulation, plan to develop a comparison joint display to compare quantitative and qualitative results about feasibility domain(s) to determine a substantiated answer to the question as to whether a study is feasible. Use the table rows to represent each major feasibility domain (or facet of a major domain). Use the table columns to represent the quantitative evidence, qualitative evidence, and overall (joint) interpretation. This table will juxtapose the quantitative and qualitative evidence for each domain so the researcher can determine the level of agreement in the evidence, including areas of corroborations and dissonance to inform decisions about about whether or not (or to what extent) the procedures are feasible. See Table 1a in Additional file 1 for an example comparison template.
3. For a mixing reason of completeness, plan to develop a synthesis joint display to synthesize complementary quantitative and qualitative information to develop a comprehensive understanding of feasibility domains in response to the study’s questions. For example, an investigator could assess the acceptability domain by mixing quantitative data from ratings of intervention satisfaction with qualitative data from interviews asking participants what they liked and did not like about the intervention. Similar to the comparison joint display, this joint display is likely organized by feasibility domains (the rows) and types of data/results (the columns). The number of columns would reflect the nature of the information that is being examined and might represent different data forms, different stakeholders, and/or different perspectives (e.g., facilitators and barriers). This table will summarize a broad range of findings about each domain to facilitate the investigator’s ability to synthesize the information and develop insights about the complexity of the feasibility of the study procedures. See Table 2 a and b in Additional file 1 for example synthesis templates.
4. For a mixing reason of explanation, plan to develop an interconnection joint display to interconnect quantitative and qualitative information to uncover differential patterns within feasibility domains and address questions about feasibility within different contexts. Start by identifying subgroups that may be important to consider in understanding feasibility within the study contexts. These subgroups might be based on location (e.g., different clinic sites), demographics (e.g., different cultural groups), quantitative measures (e.g., participants with high, medium, and low adherence), or qualitative types (e.g., participants described as fearful, apathetic, and optimistic from thematic analysis). Create a table that cross-tabulates the different groups (the rows) with the group’s corresponding quantitative and qualitative results (the columns). This table can then uncover patterns among the different groups and provide new insights that help to explain what was feasible for whom. See Table 3a in Additional file 1 for an example interconnection template.

### Planning consideration 5: prepare to draw meta-inferences about feasibility from the integrated data

The final mixed methods planning consideration is for investigators to prepare to draw conclusions from the integrated quantitative and qualitative results to form meta-inferences. Meta-inferences require investigators to interpret and consider implications for what has been learned from the combination of quantitative and qualitative results from their studies [28]. Investigators should examine the quantitative, qualitative, and mixed methods data collected to draw conclusions and meta-inferences about overall feasibility in pilot feasibility studies where not all feasibility domains were selected for mixing. Investigators are encouraged to look for both consistencies and inconsistencies within the different sets of results and consider both as opportunities for learning and gaining insight about the study’s research questions and identifying implications for a future trial [27, 54]. If inconsistencies are discovered, investigators are encouraged to revisit their data and results and attempt to fully understand divergent results, which could lead to deeper insights about important nuances in the feasibility and acceptability of an intervention and/or study procedures.

These meta-inferences should inform the future trial’s intervention design, outcome measures, and/or methodology. Meta-inferences drawn from the combined quantitative and qualitative results may be ideally suited to provide nuanced insights into the modifications that are required to optimize the intervention and study procedures for the targeted participants or the areas that are in need of careful monitoring. However, a potential pitfall is for pilot study investigators to lose track of these insights throughout the pilot study process, particularly if the pilot study involves multiple iterations of testing and refining. Investigators can address this by developing an audit trail or log where they record each meaningful interpretation that occurs during the pilot study process and the corresponding implications for the future trial. Detailed records can be very useful for conveying to reviewers how the full trial is informed by the pilot study results.

Investigators should plan to draw meta-inferences about the feasibility of the future trial by considering the following steps:

1. Plan to interpret the quantitative, qualitative, and mixed methods results to draw conclusions and meta-inferences about the feasibility of the intervention and study procedures. When interpreting the mixed methods results, consider the insights gained from comparing, synthesizing, and interconnecting the quantitative and qualitative results.
2. Consider the implications of the meta-inferences for improving the intervention parameters. For example, the implications might suggest modifications for the intervention components, mode of delivery, intervention duration, cultural sensitivity of intervention materials, or role of supporting clinic personnel.
3. Consider the implications for optimizing the outcome measures. For example, the implications might suggest adjusting the frequency or duration of the measures, adding measures for previously unanticipated outcomes, or improving the appropriateness of the planned measures for participants.
4. Consider the implications for modifying the trial’s methodology. Possible modifications might include refining recruitment materials, using recruiters of similar cultural background as the target participants, selecting the most appropriate trial design (e.g., cluster randomized trial vs. non-randomized stepped wedge design based on stakeholder preferences), or refining the timing of quantitative and qualitative data collection.
5. Maintain an audit trail of modifications identified throughout the pilot study process, including the supporting evidence and meta-inferences that formed the basis of the conclusions. Use this audit trail when planning the next study as well as when describing in grant applications how the subsequent trial is informed by what was learned in the pilot feasibility study.

## Conclusions

We identified a set of five planning considerations with specific steps for using mixed methods to optimize what can be learned from pilot feasibility studies to plan intervention trials. These planning considerations facilitate an investigator’s ability to successfully design a rigorous mixed methods approach that will achieve nuanced insights through meaningful integration in response to the pilot feasibility study’s goals. We encourage investigators to use these planning considerations as models for their own study planning as well as to spur their creativity in combining methods to address their feasibility questions. Although we have described the considerations in discrete and linear steps, investigators should keep in mind that study planning is a complex and iterative process, and planning considerations should be selected and applied in an order that is most appropriate for a particular pilot feasibility study. Furthermore, investigators should be flexible and responsive while conducting a mixed methods pilot feasibility study. Our methodological guidance aims to facilitate investigators’ ability to mix methods, but the planning should not restrict the possibility of following up on unexpected feasibility issues that may be uncovered during the pilot study implementation.

Pilot feasibility studies provide an important function within intervention research by helping investigators optimize their intervention and study procedures for future trials that will determine the efficacy and effectiveness of the intervention. Mixed method approaches have the potential to enhance a pilot study’s ability to generate nuanced and useful information about the feasibility of an intervention and the trial procedures, which can inform any needed modifications to optimize a future definitive trial. Challenges to a rigorous mixed methods approach include trying to apply mixed methods to all feasibility questions, collecting too much data that is difficult to interpret, and missing opportunities to gain new insights from the combination of different data and results. Through careful planning, investigators can address these challenges by identifying one or more key feasibility domains and reasons for mixing methods, focusing data collection sources and timing of data collection for the key domain(s) of interest, and using joint displays to help with integrative analyses and drawing meta-inferences.

Collectively, the planning considerations described in our guidance provide a practical approach to conceptualizing the elements required to optimize the use of mixed methods and achieve integrated insights. The key is for investigators to design a rigorous and focused mixed methods approach that not only includes both quantitative and qualitative data effectively and meaningfully but also integrates the quantitative and qualitative information in response to the pilot study’s specific feasibility questions. Our guidance can help investigators to consider the range of decisions needed at the study conceptualization stage to achieve a mixed methods approach and enhanced insights that will lead to the development of a future intervention trial.

## Supplementary Information

**Additional file 1.**
