# Supplementary material for: Applying mixed methods to pilot feasibility studies to inform intervention trials
Source: Pilot Feasibility Stud. 2022 Sep 26;8:217. doi: 10.1186/s40814-022-01178-x (PMC9511762; doi:10.1186/s40814-022-01178-x)
Supplement: Supplementary file 1 — Additional file 1. [file 40814_2022_1178_MOESM1_ESM.docx]

**Template Examples for Three Types of Joint Displays for Mixed Methods Pilot Studies**

This file provides the mixing reasons, example mixed methods integration questions, and example templates for comparison, synthesis, and interconnection joint displays developed for mixed methods pilot studies. The examples are for illustration purposes, and investigators are encouraged to refine the organization and content of their joint displays in light of their pilot study questions and data. To assist with applying these templates, we also provide citations to examples of these joint displays found in published literature.

**(1) Comparison Joint Display**

*Reason for mixing methods*:

- Triangulation (compare quantitative and qualitative results to determine to what extent and in what ways a domain of concern is and is not feasible)

*Example mixed methods integration questions*:

- To what extent and in what ways is [domain of concern] feasible?
- To what extent do the quantitative and qualitative results agree about the feasibility of the domain?
- To what extent and in what ways is [domain of concern] feasible when quantitative and qualitative results are compared?

*Example Template:*

**Table 1a. Joint display comparing quantitative and qualitative results about [feasibility domain(s)]**

| **Domains** | **Quantitative Results** | **Qualitative Results** | **Mixed Methods Interpretation** |
| --- | --- | --- | --- |
| *Feasibility domain #1:  -Primary domain and/or subdomains* | *Summary of quantitative evidence about the extent of the domain’s feasibility (yes, no, partial)* | *Summary of qualitative evidence about the extent of the domain’s feasibility (yes, no, partial)* | *(1) Determine points of agreement and disagreement between the quantitative and qualitative evidence for this domain and explain any points of disagreement*  *(2) Interpret the extent to which this domain is feasible (e.g., yes, no, partial) based on the combined evidence and benchmark criteria* |
| *Feasibility domain #2:*  *-Primary domain and/or subdomains* | *Summary of quantitative evidence about the extent of the domain’s feasibility (yes, no, partial)* | *Summary of qualitative evidence about the extent of the domain’s feasibility (yes, no, partial)* | *(1) Determine points of agreement and disagreement between the quantitative and qualitative evidence for this domain and explain any points of disagreement*  *(2) Interpret the extent to which this domain is feasible (i.e., yes, no, partial) based on the combined evidence and benchmark criteria* |
| ** Add rows as needed for additional feasibility domains* | | | |

*Published examples:*

- Tables 4, 5, and 6 from Leiler, A., Wasteson, E., Holmberg, J., & Bjärtå, A. (2020). A pilot study of a psychoeducational group intervention delivered at asylum accommodation centers-A mixed methods approach. *International Journal of Environmental Research and Public Health*, *17*(23), 8953. <https://doi.org/10.3390/ijerph17238953>
- Tables 4, 5, and 6 from Pedersen, M., Bennich, B., Boateng, T. et al. (2022). Peer-mentor support for older vulnerable myocardial infarction patients referred to cardiac rehabilitation: single-arm feasibility study. *Pilot Feasibility Stud* **8**, 172. <https://doi.org/10.1186/s40814-022-01141-w>

**(2) Synthesis Joint Display**

*Reason for mixing methods*:

- Completeness (synthesize quantitative and qualitative results to understand the complexity of a feasibility domain and barriers that need to be addressed)

*Example mixed methods integration questions*:

- What is the feasibility in terms of [domain of concern] and what barriers need to be addressed?
- How do the quantitative and qualitative results complement each other for understanding the feasibility of [the domain of concern]?

*Example Templates:*

**Table 2a. Joint display synthesizing quantitative and qualitative results about facilitators and barriers for [feasibility domain(s)]**

| **Domain** | **Facilitators** | **Barriers** | **Mixed Methods Interpretation** |
| --- | --- | --- | --- |
| *Feasibility domain #1  -Primary domain and/or subdomains* | *Summary of quantitative and qualitative results* | *Summary of quantitative and qualitative results* | *Develop a comprehensive understanding of the domain based on the synthesis of information and procedures that need to be addressed* |
| *Feasibility domain #2  -Primary domain and/or subdomains* | *Summary of quantitative and qualitative results* | *Summary of quantitative and qualitative results* | *Develop a comprehensive understanding of the domain based on the synthesis of information and procedures that need to be addressed* |
| ** Add rows as needed for additional feasibility domains* | | | |

**Table 2b. Joint display synthesizing quantitative and qualitative results about barriers to the [feasibility domain] for different types of participants**

| **Domain** | **Barriers Identified by Administrative Staff** | **Barriers Identified by Clinicians** | **Barriers identified by Participants** | **Mixed Methods Interpretation** |
| --- | --- | --- | --- | --- |
| *Subdomain #1* | *Summary of quantitative and/or qualitative results* | *Summary of quantitative and/or qualitative results* | *Summary of quantitative and/or qualitative results* | *Interpret the key barriers that need to be addressed based on synthesis of the evidence* |
| *Subdomain #2* | *Summary of quantitative and/or qualitative results* | *Summary of quantitative and/or qualitative results* | *Summary of quantitative and/or qualitative results* | *Interpret the key barriers that need to be addressed based on synthesis of the evidence* |
| ** Add rows as needed for additional facets of the intervention* | | | | |

*Published examples:*

- Tables 4 and 5 from Iio M, Sato M, Narita M, Yamamoto-Hanada K, Oishi T, Kishino A, Kawaguchi T, Nishi R, Nagata M, Ohya Y (2022). Development and feasibility of a mobile asthma app for children and their caregivers: Mixed methods study. *JMIR Form Res,* 6(5):e34509. <https://doi.org/10.2196/34509>
- Table 6 from Qu L, Chen H, Miller H, Miller A, Colombi C, Chen W, Ulrich DA (2022). Assessing the satisfaction and acceptability of an online parent coaching intervention: a mixed-methods approach. *Front. Psychol*, 13:859145. <https://doi.org/10.3389/fpsyg.2022.859145>

**(3) Interconnection Joint Display**

*Reason for mixing methods*:

- Explanation (Interconnect quantitative and qualitative results to uncover how and why feasibility differs for subgroups or contexts of interest)

*Example mixed methods integration questions*:

- Does [domain of concern] differ for [subgroups or contexts of interest] and if so, why?
- How and why do the identified [subgroups or contexts] differ in terms of their quantitative assessments and qualitative experiences of the feasibility domain(s)?

*Example Template:*

**Table 3a. Joint display of quantitative and qualitative differences in [subgroups or contexts] for [feasibility domain(s)]**

| **Subgroups** | **Quantitative Results** | **Qualitative Results** | **Mixed Methods Interpretation** |
| --- | --- | --- | --- |
| *Subgroup #1  (based on context, quantitative results, and/or qualitative findings)* | *Summary of key statistics for sample in subgroup #1* | *Summary of key themes and perspectives for sample in subgroup #1* | *Interpret how and why subgroup #1 differs from others* |
| *Subgroup #2  (based on context, quantitative results, and/or qualitative findings)* | *Summary of key statistics for sample in subgroup #2* | *Summary of key themes and perspectives for sample in subgroup #2* | *Interpret how and why subgroup #2 differs from others* |
| ** Add rows as needed for additional subgroups or contexts* | | | |

*Published example:*

- Table 4 from Bradt J, Potvin N, Kesslick A, Shim M, Radl D, Schriver E, Gracely EJ, & Komarnicky-Kocher LT (2015). The impact of music therapy versus music medicine on psychological outcomes and pain in cancer patients: a mixed methods study. *Support Care Cancer* 23, 1261-1271. <https://doi.org/10.1007/s00520-014-2478-7>
